# Human sweat monitoring using polymer-based fiber

**DOI:** 10.1038/s41598-019-53677-2

**Published:** 2019-11-21

**Authors:** Taekyung Lim, Youngseok Kim, Sang-Mi Jeong, Chi-Hyeong Kim, Seong-Min Kim, Sang Yoon Park, Myung-Han Yoon, Sanghyun Ju

**Affiliations:** 1 Department of Physics, Kyonggi University, Suwon, Gyeonggi-Do 16227 Republic of Korea; 2 School of Materials Science and Engineering, Gwangju Institute of Science and Technology, 123 Cheomdan-gwagiro, Buk-gu, Gwangju 61005 Republic of Korea; 3 Advanced Institutes of Convergence Technology, Seoul National University, Suwon-si, Gyeonggi-do 16229 Republic of Korea

**Keywords:** Biomedical engineering, Electrical and electronic engineering

## Abstract

Lightweight nano/microscale wearable devices that are directly attached to or worn on the human body require enhanced flexibility so that they can facilitate body movement and overall improved wearability. In the present study, a flexible poly(3,4-ethylenedioxythiophene):poly(styrenesulfonate) (PEDOT:PSS) fiber-based sensor is proposed, which can accurately measure the amount of salt (i.e., sodium chloride) ions in sweat released from the human body or in specific solutions. This can be performed using one single strand of hair-like conducting polymer fiber. The fabrication process involves the introduction of an aqueous PEDOT:PSS solution into a sulfuric acid coagulation bath. This is a repeatable and inexpensive process for producing monolithic fibers, with a simple geometry and tunable electrical characteristics, easily woven into clothing fabrics or wristbands. The conductivity of the PEDOT:PSS fiber increases in pure water, whereas it decreases in sweat. In particular, the conductivity of a PEDOT:PSS fiber changes linearly according to the concentration of sodium chloride in liquid. The results of our study suggest the possibility of PEDOT:PSS fiber-based wearable sensors serving as the foundation of future research and development in skin-attachable next-generation healthcare devices, which can reproducibly determine the physiological condition of a human subject by measuring the sodium chloride concentration in sweat.

## Introduction

Wearable healthcare electronics are health monitoring devices that can be worn directly on the human body or incorporated into wearable textiles. Such a device can facilitate periodical monitoring of a user’s physiological states in daily life. Several types of wearable healthcare electronics already exist in the commercial market—for instance, smartwatches and wrist bands, which can record biological signals (e.g., heart rate) and/or transmit the information to medical equipment, smartphones, etc. Recently, more sophisticated wearable electronics have been proposed by researchers in the form of skin-attachable and fabric-incorporated circuits^1–10^. In particular, there exist many studies on active materials and healthcare devices for sensing lactate, glucose, uric acid, or cortisol in sweat, tears, saliva, or interstitial fluid from human body. Among them, healthcare devices monitoring analytes in human sweat have drawn much attention for practical biomedical applications because sweat can be easily captured in daily life by wearable devices directly mounted on human skin.

Sweat plays an important role in the homeostasis of human body. This is because thermoregulation is achieved by sweating through evaporative cooling, whereas a certain portion of body waste and minerals are discharged via sweating. However, in the case of excessive loss of sweat, human body needs to maintain water and minerals at an appropriate level. Especially, loss of a large amount of sodium chloride through sweat may cause hyponatremia and deleterious physiological conditions^11,12^. Thus, it is very important to avoid dehydration by frequently checking the amount of salt (mainly, sodium chloride) in the body. In particular, wearable devices with the capability of monitoring the salt level in body fluids can be potentially useful to individuals experiencing severe sweating—for example, athletes during vigorous exercises, workers in high-temperature environments, visitors in tropical regions, etc. Furthermore, human health condition can be evaluated by monitoring the constituents of sweat as exemplified by fully integrated wearable sensor arrays^13^, graphene-based electrochemical devices^14^, and chemical-electrophysiological hybrid bio-sensing systems^15^. Nonetheless, most of these devices, which typically employ thin-film-based active materials for sensing, not only require complex device architectures and manufacturing processes but also show fragility against mechanical deformation.

In this study, we proposed an approach for measuring aqueous salt concentration using a flexible single-strand fiber composed of poly(3,4-ethylenedioxythiophene):poly(styrenesulfonate) (PEDOT:PSS). First, mechanically ductile PEDOT:PSS fibers were manufactured via a simple wet-spinning process and their electrical/mechanical properties were examined. The observed changes in the conductivity of PEDOT:PSS fibers were investigated depending on the concentration of aqueous sodium chloride solution in contact with fiber. Finally, a simple but robust wearable sensor based on a flexible PEDOT:PSS fiber was proposed to detect the salt concentration in the sweat directly discharged from human body.

## Results and Discussion

**Figure 1:**
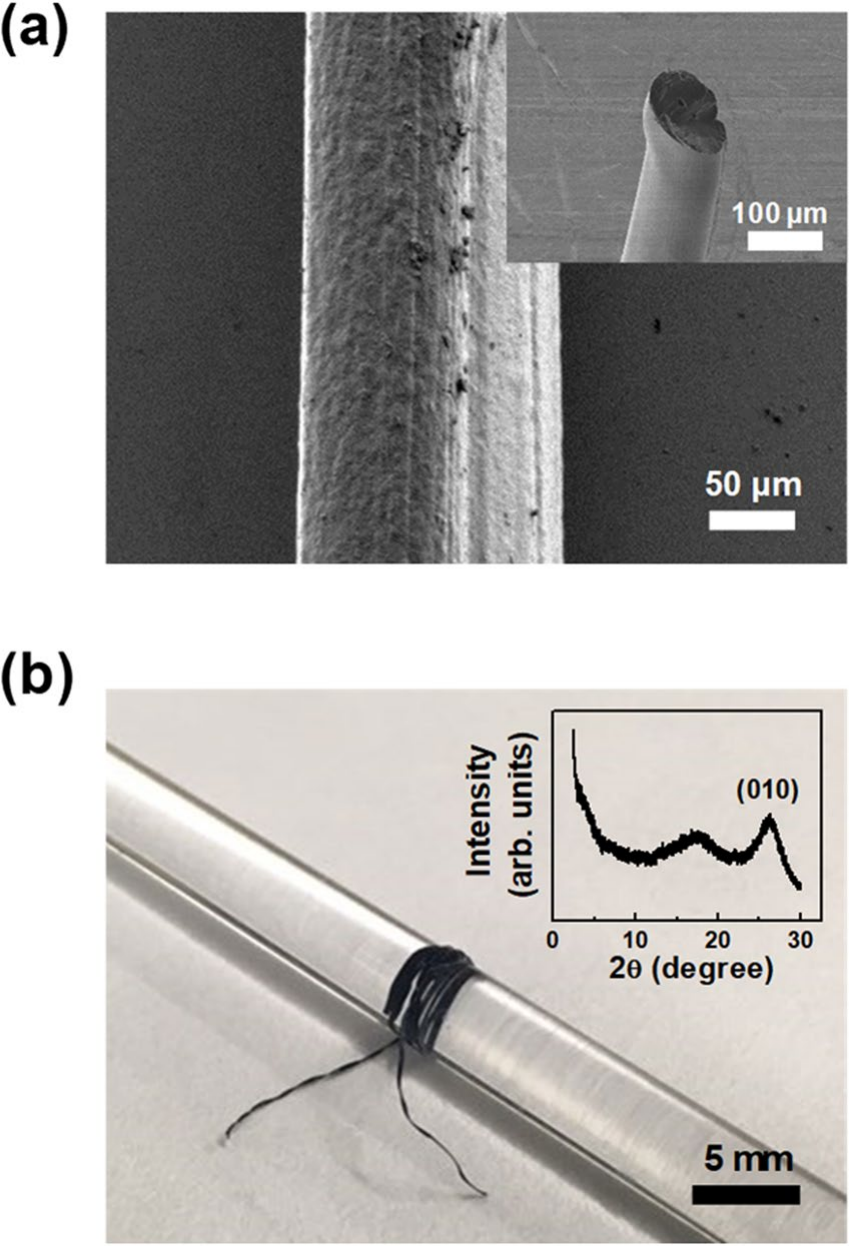
(**a**) FE-SEM image of the fabricated PEDOT:PSS fiber. All processes are compatible with the conventional wet spinning steps of coagulation, rinsing, and drying. (**b**) Photograph of spooled crystallized PEDOT:PSS fibers on the glass rod. The inset shows the XRD spectrum of the representative PEDOT:PSS fiber.

Figure 1(a) shows an FE-SEM image of the fabricated PEDOT:PSS fibers using the conventional wet-spinning method. The PEDOT:PSS is a very successful conducting polymer, made by aqueous oxidative polymerization of the monomer 3,4-ethylenedioxythiophene (EDOT) in the presence of PSS chains as a template polymer. Conductive and hydrophobic PEDOT oligomers are bounded along an insulating and hydrophilic PSS chain by electrostatic interaction between PEDOT and a positive charge, and PSS and a negative charge^16^. The PEDOT:PSS complex is dispersed in water in micro-gel form at a size of 30–50 nm. When the water in PEDOT: PSS solution is evaporated, adjacent microgels in PEDOT: PSS solution are H-bonded to each other to form a gel network^17^. In the formation of a fiber-shaped PEDOT:PSS solid in a sulfuric acid coagulation bath, PEDOT oligomers and PSS chains reacted with two types of ions, HSO_4_^–^ and H^+^, generated by the dissociation of sulfuric acid in water. In particular, when H^+^ ion and PSS chains with negative charge are reacted and become neutralized, electrostatic interactions between PEDOT oligomers and PSS chains are weakened. As a result, the free-PSS chains, which were not affected by the PEDOT oligomers, were removed from the fiber-shaped PEDOT:PSS solid^18^. In this process, the proportion of conductive PEDOT oligomers is higher than that of the insulating PSS chains in the fiber-shaped PEDOT:PSS solid, and the PEDOT networks with strong π-π stacking nature become dense. As a result, highly crystalline and conductive PEDOT:PSS fibers can be obtained. After coagulation, the resultant structures were rinsed with acetone and DI water, resulting in water-stable crystallized PEDOT:PSS fibers. Moreover, crystallized PEDOT:PSS fibers exhibited high mechanical strength because of their crystallized molecular structure, which enabled them to be wound on a glass rod with uniformly defined circular fibers. The average diameter of the PEDOT:PSS fibers was 149 ± 10 µm. The crystallinity was estimated based on the results of an XRD spectrum, which clearly depict the peak of the π–π stacking arrangement of the PEDOT chains ((010) direction), as shown in the inset of Fig. 1(b).

**Figure 2:**
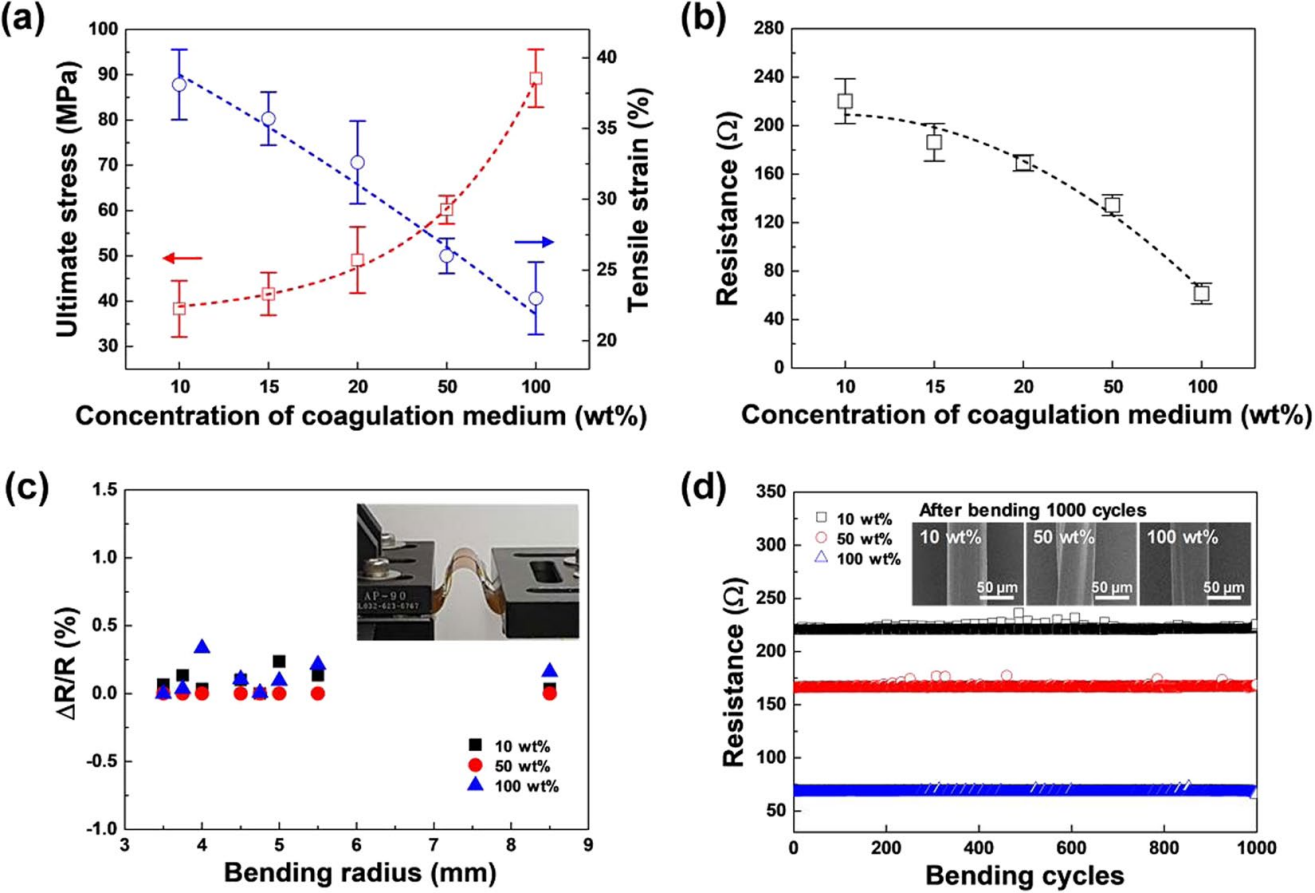
(**a**) Ultimate stress and tensile strain, (**b**) resistance, (**c**) resistance dependent on the degree of bending, and (d) changes in resistance and the FE-SEM images (1000 bending cycles) of the PEDOT:PSS fibers.

The mechanical properties of the fibers, such as strength, elongation, elasticity, and modulus, are the predominant parameters for realizing practical wearable sensors, because the degree of comfort is highly dependent on the strength and stretchability of the resulting textile material. Figure 2(a) displays the results for the ultimate stress and tensile strain of the fabricated PEDOT:PSS fibers (10, 15, 20, 50, and 100 wt%) as a function of sulfuric acid content in the coagulation process. The average values of the ultimate stress and tensile strain were determined by measuring the strain-stress curves a total of five times. This ultimate stress parameter exhibited an increase from approximately ~38 to ~89 MPa, with an increase in the concentration of sulfuric acid. Conversely, the tensile strain gradually decreased from approximately ~38 to ~23%. The ultimate stress and tensile strain values of the polyester and PU fibers fabricated by using the conventional electro, wet, and melt-spinning methods are 23–250 MPa/11–330% and 6–60 MPa/120–750%, respectively^19–21^. The ultimate stress of PEDOT:PSS fiber was within the average range of the previously reported results, but the tensile strain value of PEDOT:PSS fiber was slightly lower. As the concentration of sulfuric acid increases, the stiffness and the elongation of the PEDOT:PSS fiber increases and decreases, respectively, indicating that the rigid property of the PEDOT oligomer by the strong π-π stacking nature is more expressed than the coil-like property of the PSS at the high concentration of sulfuric acid. This is because the higher the sulfuric acid concentration is, the more free-PSS chains are removed during the wet-spinning process. As a result, the high concentration of sulfuric acid caused the PEDOT:PSS chains to be highly aligned in the fiber. The electrical conductivity is also an important parameter for sensors because the sensitivity of a sensor, especially a two-probe-resistive sensor, strongly relies on the relative conductivity of the sensing and target materials. Therefore, we measured the electrical resistance of the PEDOT:PSS fibers using the four-probe method, as depicted in Fig. 2(b). The electrical conductivity was determined to monotonically increase with sulfuric acid concentration in the coagulation bath. Since the crystallinity of the PEDOT:PSS film is related to improvement in electrical conductivity, the resultant data reveal that the increase in conductivity was mainly caused by the enhanced fiber crystallinity, which is in excellent agreement with the corresponding mechanical property^22^. We also measured the dependence of the resistivity on the degree of bending (Fig. 2(c)). It is worth noting that we did not observe a noticeable change in the conductivity, even for high degrees of bending (bending radius of approximately 0.35 cm). The bending test was performed 1000 times to evaluate the durability of the PEDOT:PSS fibers (Fig. 2(d)). During the test, we measured the electrical resistance under both straight and bending conditions. The bending radius was maintained at 0.35 cm throughout the bending tests. As shown in the figure, the electrical properties did not change during bending. The insets in Fig. 2(d) show the FE-SEM images measured after 1000 cycles of bending testing of the PEDOT:PSS fibers with three different sulfuric acid concentrations (10, 50, and 100 wt%). After repeated bending, no mechanical damage appeared on the fiber surface. This indicates that the PEDOT:PSS fibers prepared in this study are suitable for application in wearable devices. By considering the mechanical properties and conductivity, as shown in Fig. 2, the PEDOT:PSS fiber (10 wt%) was selected because it exhibited high flexibility, which is suitable for applications related to wearable electronics.

**Figure 3:**
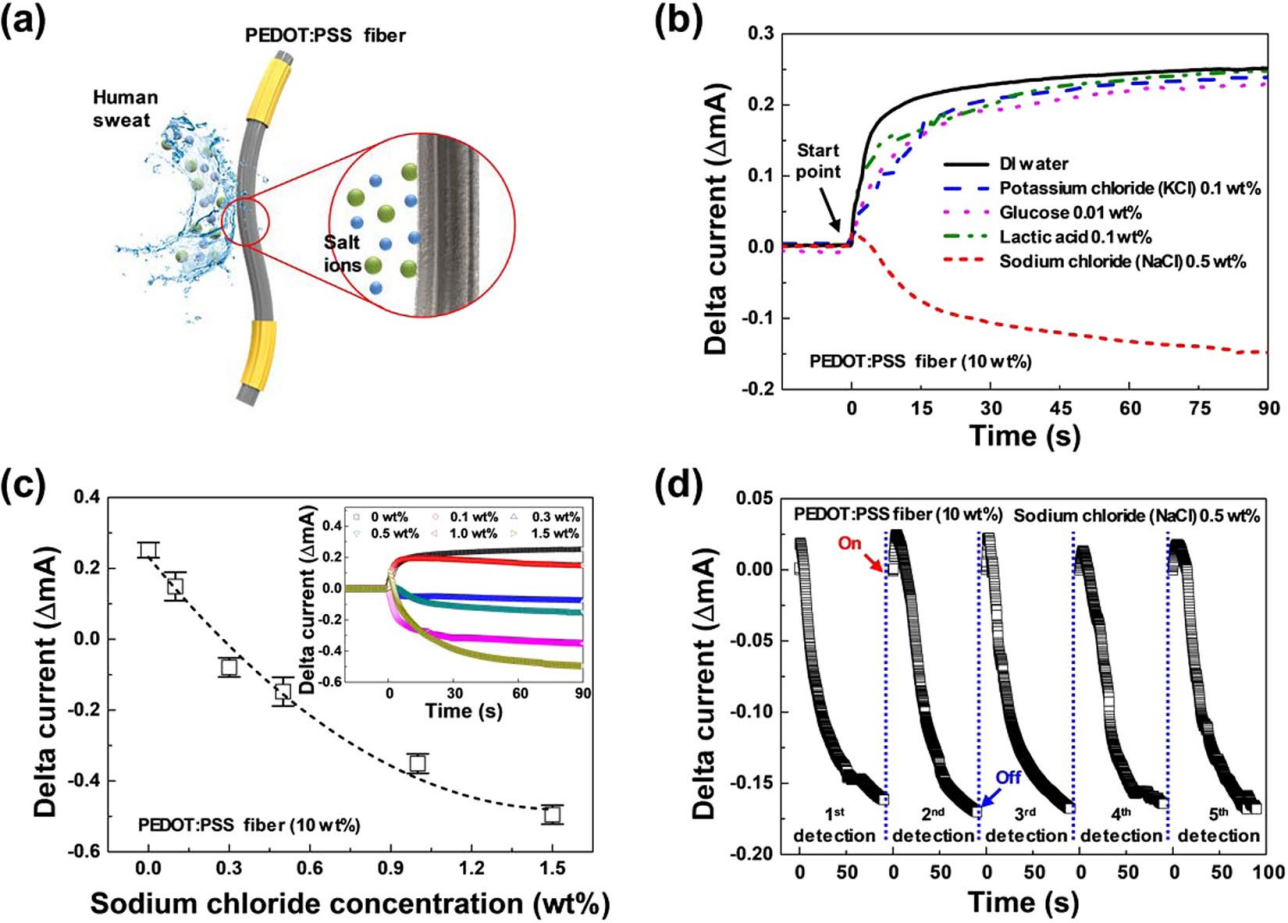
(**a**) Schematic diagram for the formation of the PEDOT:PSS fiber. The PEDOT:PSS fiber was exposed to artificial sweat between the two electrodes. (**b**) Current versus time for the PEDOT:PSS fiber (10 wt%) under salt ions (potassium chloride (KCl) 0.1 wt%, glucose 0.01 wt%, lactic acid 0.1 wt%, and NaCl 0.5 wt%) in water. (**c**) Changes in current depending on the concentration of sodium chloride in water. The columns and error bars represent the average and standard deviation of the measured currents, respectively. (**d**) Repeated time-dependent response of the device (five cycles) based on sodium chloride in water (0.5 wt%).

Figure 3(a) shows a schematic diagram of the experiment for measuring salt ions using the PEDOT:PSS fiber under artificial or human sweat. In order to form two-terminal electrodes, the 500 nm-thick aluminum was deposited on both ends of the fiber at a distance of 10 mm. Although the content of human sweat varies depending on the individual or health condition, sweat usually contains water, ions (Na^+^, Cl^−^, K^+^, NH_4_^+^), small molecules (glucose, lactic acid, urea, and ethanol), and small proteins (neuropeptides and cytokines). For artificial sweat, 0.5% of NaCl, 0.1% of KCl, 0.1% of lactic acid, and 0.01% of glucose were each added as weight/volume ratios in deionized water. Figure 3(b) shows an interesting trend in which the current flowing through the PEDOT:PSS fiber (10 wt%) rapidly increased when immersed in pure water and in DI water with 0.1% of KCl, with 0.1% of lactic acid, and with 0.01% of glucose solution. Since the concentration of KCl, lactic acid, and glucose solution was low, the current changes of the PEDOT:PSS fiber (10 wt%) was very small as the current change of the pure water. However, it decreased for the 0.5% NaCl solution in DI water. Considering that holes are the dominant carrier type generated via PSS doping, with respect to flow through the PEDOT interchain networks, polar solvent (i.e., DI water) can enhance hole mobility by the attenuation of the electrostatic interaction between PEDOT and PSS^23^. Additionally, ions and small molecules had little effect on hole mobility of PEDOT chains for the 0.1% KCl, 0.1% lactic acid, and 0.01% glucose cases, while the 0.5% NaCl solution provided an exception. Ions or small molecules were not found to migrate as much as Na^+^ into the space between PEDOT oligomers or PSS chains, which strongly interact in the fiber because the sizes of Cl^−^ (ionic radii, 0.181 nm), K^+^ (ionic radii, 0.133 nm), lactic acid, and glucose were larger than Na^+^ (ionic radii, 0.097 nm), and the concentration of NaCl in sweat was higher than other compounds^24^.

Figure 3(c) shows the current changes when six different concentrations of NaCl in water (0, 0.1, 0.3, 0.5, 1.0, and 1.5 wt%) come into contact with the surface of the PEDOT:PSS fiber (10 wt%). As shown in the inset, the PEDOT:PSS fiber was in contact with the designated sodium chloride in water until the current level was stabilized. As can be observed, a different current was obtained for each sodium chloride concentration. The concentration of sodium chloride in water at 0 wt% shows a maximum current intensity of approximately 2.5 × 10^−4^ A. Similarly, the concentrations of 0.1, 0.3, 0.5, 1.0, and 1.5 wt% exhibit currents of approximately 1.5 × 10^−4^, –0.8 × 10^−4^, –1.5 × 10^−4^, − 3.5 × 10^−4^, and –5.0 × 10^−4^ A, respectively. As a result, the increase in sodium chloride concentration caused an increased change in the current with high output signal over 600 μA/dec. in response to the NaCl concentration range in human sweat owing to the high channel cross-sectional area and electrical conductivity. Note that the conventional ion concentration sensors which are typically based on three-terminal devices exhibit relatively lower output signal than ours based on two-terminal single fiber (see the Table 1)^25,26^. The current was measured at five different points for each sample, and the average and standard deviation were calculated and are represented as columns and error bars, respectively. Due to the increase in sodium chloride concentration (0, 0.1, 0.3, 0.5, 1.0, and 1.5 wt%), the Δ*I*_*ds*_ values decrease linearly to (2.51 ± 0.21) × 10^−4^, (1.49 ± 0.40) × 10^−4^, (−0.80 ± 0.27) × 10^−4^, (−1.48 ± 0.41) × 10^−4^, (–3.51 ± 0.28) × 10^−4^, and (−4.95 ± 0.27) × 10^−4^ A, respectively. Figure 3(d) shows the repeated time-dependent response of the device when exposed to sodium chloride (0.5 wt%). As shown in the figure, the current decreases to approximately −1.7 × 10^−4^ A after NaCl (0.5 wt%) is placed in water on the device. The current changes were measured repeatedly (up to five cycles), indicating that the responsive property is highly reproducible (Fig. 3(d)). Note that as the diameter of the PEDOT: PSS fiber increased, the amount of current change by NaCl in water solution decreased. Moreover, since the sensing mechanism is based on the ionic diffusion through the PEDOT:PSS chains of cations in electrolyte, the reaction rate (time constant) could be decreased (increased) to fully dedope/dope the inner PEDOT:PSS chains in larger diameter.

**Table 1:** Performance comparison with previous NaCl detecting devices.

References	Device platform	Supplying voltage	Number of nodes	Output signal
25	Thin film	V_G_ = 0 V	3	~50 μA/dec.
		V_D_ = −0.7 V		
26	Thin film	V_G_ = 0.4 V	3	~300 μA/dec.
		V_D_ = −0.1 V		
This work	**Fiber**	**V** **=** **0.5 V**	**2**	**~600** **μA/dec**.

Two reference papers don’t show the exact performance parameters, so that those values were extracted from the representative figures.

The PSS chain constituting the PEDOT:PSS fiber binds several PEDOT oligomers by electrostatic interaction. In the PEDOT:PSS fiber, the PSS-rich portion intertwines with only PSS moieties and the PEDOT-rich portion is located between the PEDOT oligomers and PSS moieties^27^. During the wet-spinning process, the PSS-rich portion of the PEDOT:PSS fiber is separated from each other due to the coulombic repulsion between the PSS negative charges, stretching the PSS moiety. In addition, the PEDOT-rich portion is strongly bound to each other through the π-π stacking nature between the inter-chains. When the PEDOT:PSS fiber comes into contact with the salt-free water, the negative charges of the PSS-rich portion are stabilized by water as a polar solvent because the coulombic repulsion between the PSS negative charges decreases. Also, in the PEDOT-rich portion, the electrostatic interaction between PEDOT oligomers and PSS chains decreases, thereby increasing the mobility of the holes transporting through the PEDOT interchain networks. On the other hand, when the PEDOT:PSS fiber comes into contact with the NaCl solution, Na^+^ and Cl^−^ ions dissociated by water electrostatically interact with PSS negative charges. Therefore, the volume of the insulating PSS-rich portion increases due to the coulombic repulsion between the negative charges of PSS chains or the Na^+^ ions interacting with the negative charge of the PSS chains. As a result, the distance between the PEDOT inter-chains with π-π stacking increases^28^. In other words, when the PEDOT:PSS fiber comes into contact with the NaCl solution at a concentration that can cause a sufficient coulombic reaction force in PSS chains, the PEDOT networks connected by the π-π stacking interaction is partially destroyed to reduce the electrical conductivity through the PEDOT networks. On the other hand, when the PEDOT:PSS fiber is dried separately from the solution, the volume of the PSS-rich portion is reduced, restoring the electrical conductivity to the original level by re-stacking the π-π interaction of the PEDOT inter-chains.

**Figure 4:**
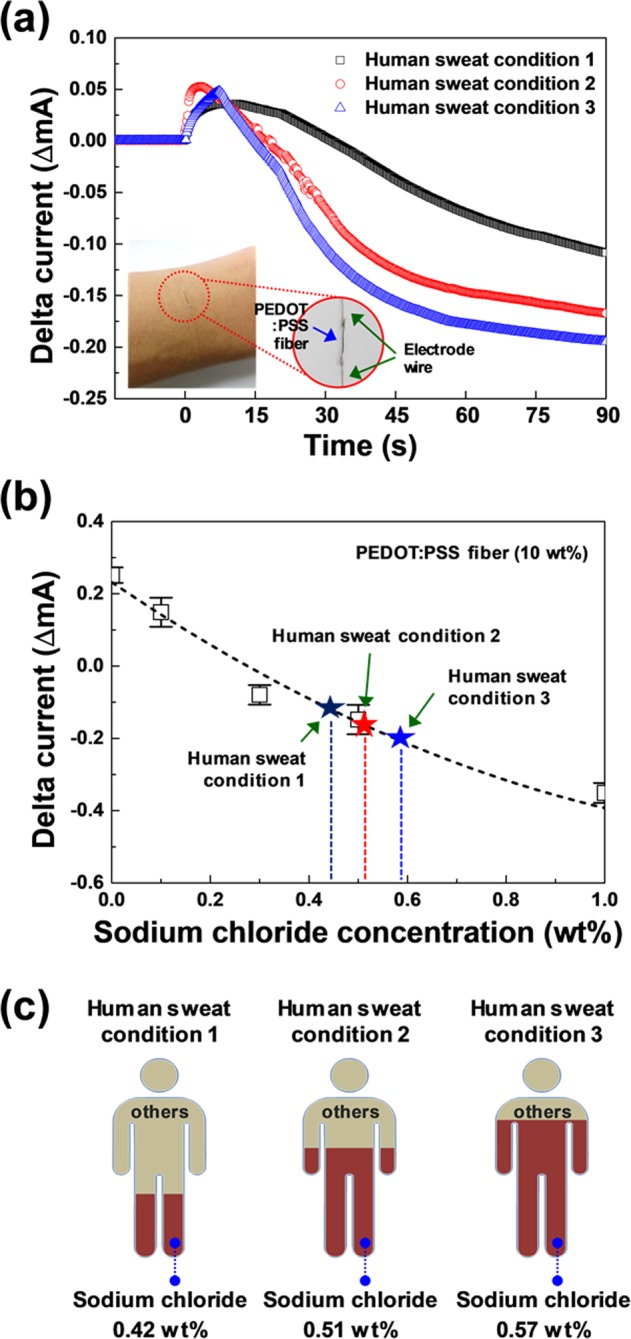
(**a**) Current versus time for the PEDOT:PSS fiber under three different conditions of sweat (condition 1, 2, and 3). Inset shows the photo image of the PEDOT:PSS fiber sensor. (**b**) Changes in the current compared with the measured data of sodium chloride in water. (**c**) Sodium chloride concentration in sweat for the three conditions.

To demonstrate an application of the fabricated flexible PEDOT:PSS fiber sensor, the sensing properties of the fabricated PEDOT:PSS fiber were examined using three different samples of human sweat (condition 1, 2, and 3). The primary component of sweat is water, which makes up 99% of this fluid, and the remaining constituents include sodium (0.4 to 1%), ammonia, potassium, magnesium, and trace amounts of glucose and lactic acid. The effect of sweat is shown in Fig. 4(a). The inset displays an image of the wire-type flexible PEDOT:PSS fiber sensor mounted on a human arm. As can be seen, a different current value was obtained for each condition of sweat. Condition 1 shows a minimum current intensity of approximately −1.09 × 10^−4^ A; similarly, conditions 2 and 3 reveal a change in the current of −1.67 × 10^−4^ and −1.95 × 10^−4^ A, respectively. The PEDOT:PSS fiber exhibits different current changes for the three human sweat samples because of the different salt content. The obtained changes in the current of the PEDOT:PSS fiber using the three human sweat samples were compared with those based on the sodium chloride concentration in Fig. 3(c). As shown in Fig. 4(b), the salt concentration can be estimated as 0.42 wt% (condition 1), 0.51 wt%, (condition 2), and 0.57 wt% (condition 3). To confirm that we can estimate the sodium chloride concentration in sweat by monitoring the current level of the PEDOT:PSS fiber, the real sodium chloride concentrations of the three human sweat samples were analyzed using an ion chromatograph and inductively coupled plasma spectrometer. Figure 4(c) shows the schematic diagram showing sodium chloride concentration in sweat according to three different conditions of human sweat. The extracted sodium chloride concentration in the human sweat samples (condition 1, condition 2, and condition 3) were 0.43, 0.54, and 0.56 wt%, respectively.

## Conclusions

A flexible PEDOT:PSS fiber sensor, which can be used to measure the concentration of salt in water, was fabricated. The PEDOT:PSS fiber was manufactured using a conventional wet-spinning process in a sulfuric acid-based coagulation bath. It had a highly crystallized structure characterized by high mechanical strength and water-immersion stability. Furthermore, the size and resistance of the resultant fiber could be controlled by adjusting the concentration of the coagulation medium. The conductivity of the PEDOT:PSS fiber increased in pure water due to the screening effect of a polar solvent (i.e., water), whereas the conductivity decreased with an increase in salt concentration due to de-doping by cations. The results of this study clearly demonstrate that the concentration of salt in sweat released from the human body can be measured using the changes in the electrical signals of the PEDOT:PSS fiber. The micro-fibrillar structures reported in this investigation do not require a complex fabrication technique, so it can be easily produced on a mass scale. As such, the developed crystalline PEDOT:PSS fibers can potentially be employed as a wearable patch-type sensor with very simple and cost-effective structures.

## Methods

The preparation of crystallized PEDOT:PSS fibers is described in this section. An aqueous solution of PEDOT:PSS (Heraeus, Clevios PH1000) was concentrated twice via the evaporation of water with a rotary evaporator (Hahnshin Scientific, HS-2005V-N) to increase its viscosity. Subsequently, the concentrated solution was slowly injected into an acid-based coagulation bath and maintained for 10 min, with the use of a syringe pump (KD Scientific, KD100) at a pumping rate of 10 mL/hr. The coagulation medium was prepared by diluting concentrated sulfuric acid (ChemiTop, 95–99%) with deionized water (DI water) to form various final concentrations (i.e., 10, 15, 20, 50, and 100 wt%). Subsequently, the resultant fibers were rinsed in acetone and DI water for 10 min, followed by drying in a vacuum oven at 120 °C for an hour. The overall morphology of each fiber was measured using field emission scanning electron microscopy (FE-SEM, JEOL, JSM-7500F), and its crystallinity was studied using X-ray diffraction (XRD, Rigaku, D/max 2500) with Cu–Kα radiation.

The mechanical properties were analyzed using a thermal mechanical analyzer (TMA, Toshiba TM2000). The fabricated PEDOT:PSS fibers were measured five times. Electrical conductivity measurements were performed based on the conventional four-probe method using a source measurement unit. Bending and mechanical durability tests were performed using a thermal mechanical multi-tester (Invisible Inc, TMM-1000).

Two electrodes made of copper wire were attached to a PEDOT:PSS fiber. The channel length of the PEDOT:PSS fiber between the two electrodes was approximately 10 mm. To prevent an electrical short-circuit between the electrodes, only the channel region of the PEDOT:PSS fiber was exposed to the sodium chloride (NaCl) in water, which consisted of a mixture of NaCl and DI water. The fabricated PEDOT:PSS fiber was immersed in six different NaCl solutions with salt concentrations of 0, 0.1, 0.3, 0.5, 1.0, and 1.5 wt%. Experiments were performed on the PEDOT:PSS fiber at an applied voltage of 0.5 V. The electrical characteristics of the fibers were measured using a semiconductor parameter analyzer (Agilent B1500A). All PEDOT:PSS fibers were utilized under a relative humidity of approximately 36.4% and an ambient temperature of approximately 24 °C.
